# The occurrence and nature of early signs of schizophrenia and psychotic mood disorders among former child and adolescent psychiatric patients followed into adulthood

**DOI:** 10.1186/1753-2000-2-30

**Published:** 2008-10-17

**Authors:** Ulf Engqvist, Per-Anders Rydelius

**Affiliations:** 1 Department of Woman and Child Health, Karolinska Institutet, Astrid Lindgren Children's Hospital, Karolinska University Hospital, SE-171 76 Stockholm, Sweden; 2 Department of Social Work, Mid-Sweden University, SE-831 25 Östersund, Sweden

## Abstract

**Background:**

This investigation was designed to characterize psychotic disorders among patients originally treated as in- and outpatients by child and adolescent psychiatric services and subsequently followed-up into mid-adulthood. The age at the first onset on symptoms, possible changes in diagnoses, early signs noted prior to or upon admission to child and adolescent psychiatric care and possible differences between patients with early- and later-onset disorder were of particular interest.

**Methods:**

The study population consisted of patients (285 in- and 1115 outpatients) born between 1957 and 1976 and admitted to and treated by child and adolescent psychiatric care units in Jämtland County, Sweden, between 1975 and 1990. The status of their mental health was monitored until 2003 using official registries and hospital records. Diagnoses based on the ICD-8 and -9 systems, which were used in Sweden from 1968–1997, converted to diagnoses according to ICD-10, which has been in use since 1997. The Comprehensive Assessment of at Risk Mental States was employed to assess the information concerning psychopathology provided by the hospital records.

**Results:**

By the end of the follow-up period 62 former child and adolescent psychiatric patients (36 females and 26 males), 4.4% of the entire study group, had received an ICD-10 diagnosis of "F20–29: Schizophrenia, schizotypal and delusional disorders" (48) and/or "F30–39: Psychotic mood disorders" (14). One-third (21) of these individuals were given their initial diagnosis of psychosis in connection with child and adolescent psychiatric care. Two of these 21 were not treated later for this disorder in general (adult) psychiatric care whereas the remaining 19 individuals were diagnosed for the same type of disorder as adults. The other 41 patients were diagnosed as psychotic only in connection with general (adult) psychiatric care. The mean age at the time of first onset of symptoms was 21.4 years (SD 6.4) and corresponding median age was 18. Behavioural changes and positive symptoms were the most frequent signs associated with a diagnosis of "F20–F29: Schizophrenia, schizotypal and delusional disorders" made during child and adolescent psychiatric care. In cases where a specific psychopathology developed later on the initial admission to child and adolescent psychiatry involved unspecified psychopathology.

**Conclusion:**

In summary, it appears that psychotic disorders are relatively uncommon among patients admitted to child and adolescent psychiatric care in Sweden. However, individuals experiencing early onset of disorders categorized as "F20–29: Schizophrenia, schizotypal and delusional disorders" may already exhibit typical symptoms upon admission to child and adolescent psychiatric care of the age of 13–17; whereas late-onset disorders it appear not be associated with any obvious signs or symptoms years before the disorder has developed fully. Finally, certain cases of psychotic disorder during adolescence seem to have been episodic.

## Background

For more than three decades, Michel Rutter and co-workers [1-3] have periodically reviewed the literature concerning relationships between childhood and adult psychopathology, with particular focus on possible mechanisms involved in the continuities and discontinuities observed between early and later psychopathology, as well as the need for systematic, prospective and long-term longitudinal investigations in this area. In Sweden, extensive knowledge concerning patients in child and adolescent psychiatry (CAP), has been obtained from such studies with 20–40-year periods of observation of various cohorts between 1928 and 2003 [4-7]. In addition, Swedish CAP patients have been examined employing cross-sectional approaches [8-10]. These investigations have been possible as a result of the long-standing Swedish practice of gathering data concerning individuals' health and social adaptation in general registries, which provide an exceptional and unique source of information for monitoring both diseases and social problems. A personal identification number assigned to each inhabitant allows data concerning individual treatment and outcome to be followed over the course of several decades.

The population of Swedish CAP patients is heterogeneous, including children who demonstrate problems at school, adjustment/behavioural symptoms and/or psychiatric problems, as well as children with psychosocial, family-related difficulties [4,11,12]. Those treated prior to 13 years of age often exhibit behavioural symptoms and difficulties with adjustment to peer groups and to school and other members of their families frequently experience psychosocial problems as well. In contrast, adolescents receiving such care appear to develop their "own" more often than do infants and school children, with less common occurrence of parallel psychosocial problems among the rest of the family. The typical CAP patient is either "a troublesome 10-year-old boy" or "a depressed 14-year-old girl" [4,8,11,12].

At least a third of all CAP patients, and more often girls than boys, are later seen again as psychiatric patients after reaching adulthood [6,7,12]. However, the correlation between the nature of the psychopathology requiring CAP care and later diagnosis as an adult is weak. Only a small group of patients require continuous care from child- to adulthood. Furthermore, the major reasons for which former CAP patients are admitted to general psychiatric (GenP) care are drug and/or alcohol addiction and/or criminality, rather than symptoms of psychiatric disorder [5,7,11,12].

### Aim of the present study

Our goal here was to obtain answers to a number of questions concerning a group of former CAP patients diagnosed during child- or adulthood as suffering from schizophrenia, schizotypal disorder, delusional disorders and/or psychotic mood disorders: At what age was the diagnosis made? Was this diagnosis later changed and, if so, in what manner? Were early signs of the disorder detectable prior to or at the time of admission to CAP care? Which CAP patients were later diagnosed as psychotic in GenP? And how did this latter group differ from those who had already received a diagnosis before the age of 18 years?

## Methods

### The study population

Jämtland County is one of Sweden's 21 counties. It is located in the western part of middle Sweden at the Swedish boarder to Norway. From 1975 – 2003, the total population has varied from 133,433 to 127,645 with a peak of 136,301 inhabitants in 1994.

All 1,420 patients born between 1957 and 1976 and admitted to in- or outpatient CAP care in Jämtland County, Sweden, during of the period 1975–1990 were initially considered for inclusion. Eight individuals not covered by the national registries and twelve who subsequently emigrated during the follows-up period were excluded, leaving a total of 1,400 former CAP patients, including 285 in- and 1,115 outpatient, or 98.6% of the original population.

These children and adolescents were referred to CAP by paediatricians or general practitioners (35%), by school or childcare personnel (22%), by social services (12%) or other authorities (2%) or else they themselves and/or their parents sought help (29%). They were all evaluated, in general treated and terminated their contact with CAP between 1975 and 1990, although certain some of the youngest patients were readmitted to such care subsequent to 1990.

### Experimental design and procedures

In 1995, a protocol for describing the patients and their histories was established. After identification of patients previously receiving CAP and/or GenP care, both within and outside Jämtland County on the basis of hospital records and linkage to the nationwide Swedish Hospital Discharge Registry (HDR), their gender, present age, reason for initial contact with CAP and/or GenP, and diagnoses, as well as any necessity for inpatient care were noted. During the periods of 1968–1996 and 1987–1996, the ICD-8 and ICD-9 systems, respectively, were employed in Sweden, prior to the introduction of ICD-10 in 1997. To allow comparisons all diagnoses based on the to ICD-8 and ICD-9 categories were converted to ICD-10 [13,14] utilizing the official conversion tables published by the Swedish National Board of Health and Welfare [15,16]. Although the Swedish Association for Child and Adolescent Psychiatry has decided to also apply the DSM system in parallel for clinical practice, obligatory ICD classification is utilized for official registration of diagnoses.

All of the 285 CAP patients admitted to the in-patient care received a diagnosis in connection with their treatment, whereas outpatients were not usually given a diagnosis in cases where their symptoms and problems were developmental in origin or a reaction to their living circumstances. Nonetheless, for 616 of these 1,115 outpatients (55%), a CAP diagnosis was recorded. In the case of GenP, both in- and outpatients received a diagnosis, so that 524 of the 531 patients (99%) later admitted to GenP had diagnoses noted in their hospital records and/or in the HDR registry. Specific evaluation of hospital records indicating a diagnosis of psychosis was performed.

Until 1995 combinations of retrospective and prospective approaches were employed, whereas thereafter only prospective methods were used until 2003. The mean observation time was 16.1 (SD 8.5) years, with a range of 12–28 years. A 20-year follow-up was available for 608 of the 1115 outpatients in our study group. Utilizing the t-test for a difference between two proportions the outcomes of these long-term follow-ups have been compared, to published data concerning the occurrence and frequencies of psychotic disorders observed in connection with the 20-year prospective follow-up of 2,164 outpatients treated at the Child Guidance Clinics in Stockholm during the period of 1953–1955 [4,5].

### Collection of data

After eliciting the required permission and ethical approval, collection of the data was initiated by examining the CAP hospital records, following which a prospective survey of number of these patients later referred to GenP care prior to 2003 was performed. Information concerning out- and inpatient GenP care in Jämtland County was obtained by examining local registries, hospital records and the nationwide Swedish Hospital Discharge Registry (HDR) corresponding. Information regarding inpatient care outside of this county was provided by the HDR (which only covers inpatient care).

The CAP hospital records of those patients who received a diagnosis of schizophrenia and/or psychotic mood disorders at any time during the follow-up were evaluated in greater detail for any early signs of possible psychosis utilizing the Comprehensive Assessment of at-Risk Mental States (CAARMS) developed by Yung and colleagues [17]. The goals of this instrument are two-fold, i.e., to assess psychopathology thought to indicate imminent development of a first-episode psychotic disorder and to determine whether an individual is at ultra-high-risk (UHR) for onset of an initial psychotic disorder. The diagnostic criteria for UHR have been refined for improved precision by researchers at the University of Melbourne [18,19] and Yale University [20], who have developed sets of criteria based on the presence or onset of one or more of the following: attenuated psychotic symptoms (ideas of reference, magical thinking, perceptual disturbance, paranoid ideation, and odd thinking and/or speech); intermittent psychotic symptoms of too short duration to meet the criteria of the Diagnostic and Statistical Manual of Mental Disorder for psychosis i.e., (symptoms which spontaneously disappear within 1 week); a first-degree family history of a psychotic or bipolar disorder; or a personal history of schizotypal personality disorder, with significant recent functional decline [21].

### Analysis of the data

The findings based on prospective data are descriptive in nature. All data analysis was performed using the SPSS for Windows, release 12.0 (SPSS Inc) software.

The chi-square and t-tests were employed to analyze differences between categorical and continuous variables, respectively, with a P-value of < 0.05 being considered statistically significant in both cases. Differences between proportions were analyzed utilizing a two-by-two cross table and Students t-test. Although this t-test is essentially not valid for making such comparisons, extensive studies have shown it to be applicable also in these respects, and, consequently, the student's t-test has been widely and successfully used for analysis of proportional data [22].

There are a number of proposals concerning how to present double-sided probabilities used to compare proportional data [23]. When employing the sum of small (or significant) p-values, all possible tables are generated within given margins, all p-values of the same size or smaller than the point probability are added together to obtain the cumulative p-value, and the resulting value is presented utilizing the notation p (O> = E|O< = E). In the case of the method of small p-values, the exact point probability for the nil hypothesis that produces the table observed is calculated first. Thereafter all possible alternative outcomes given the set conditions are generated with a computer program [22]. Since the number of calculations required with exact approaches can easily become excessive (particularly in the case of larger tables), computer programs that provide exact probabilities using the method of small p-values (such as the SPSS) sometimes extract a single sample from all of the possible alternatives and use this to calculate an exact probability value within confidence limits.

### Ethical considerations

The ethical committees of Umeå University (UM documents no. 95-051 and 99-023) and Karolinska Institutet (KI document no. 99-209) both pre-approved this study.

## Results

### The incidences of schizophrenia and psychotic mood disorders among our patients

By the end of the follow-up period 62 former CAP patients (36 women and 26 men), which is 4.4% of the entire study population of 1,400, had received an ICD-10 diagnosis of "F20–29: Schizophrenia, schizotypal and delusional disorders" (48 patients) and/or "F30–39: Psychotic mood disorders" (14 patients). The gender distribution among these patients was similar to that among the remainder, who had not been diagnosed as experiencing a psychosis. The various diagnostic groups are presented in Table 1.

**Table 1:** Diagnoses of psychosis recorded in connection with CAP and GenP care of our group of patients

**Diagnosis**	**Number diagnosed in connection with CAP**		**Number diagnosed in connection with GenP**	
	**Boys**	**Girls**	**Men**	**Women**
All	8	13	23	32

***Schizophrenia, schizotypal and delusional disorders***	***7***	***9***	***17***	***26***

F20.0 Paranoid schizophrenia	0	0	1	0

F20.1 Hebephrenic schizophrenia	0	0	0	1

F20.2 Catatonic schizophrenia	0	0	0	1

F20.3 Undifferentiated schizophrenia	1	0	1	8

F20.5 Residual schizophrenia	0	0	1	0

F20.8 Other schizophrenia	0	0	0	1

F20.9 Schizophrenia, unspecified	0	0	5	5

F21 Schizotypal disorder	1	2	1	0

F22.0 Delusional disorder	0	0	1	0

F23.0 Acute polymorphic psychotic disorder without symptoms of schizophrenia	0	0	0	1

F23.9 Acute and transient psychotic disorder, unspecified	5	7	2	2

F25.0 Schizoaffective disorder, manic type	0	0	0	2

F25.1 Schizoaffective disorder, depressive type	0	0	2	1

F25.2 Schizoaffective disorder, mixed type	0	0	2	1

F25.9 Schizoaffective disorder, unspecified	0	0	0	1

F29 Unspecified nonorganic psychosis	0	0	1	2

***Psychotic mood disorders***	***1***	***4***	***6***	***6***

F30.8 Other manic episodes	0	4	0	1

F31.0 Bipolar affective disorder, current episode hypomanic	0	0	0	1

F31.2 Bipolar affective disorder, current episode manic with psychotic symptoms	0	0	1	1

F31.3 Bipolar affective disorder, current episode mild or moderate depression	0	0	0	1

F31.5 Bipolar affective disorder, current episode severe depression with psychotic symptoms	1	0	0	0

F31.6 Bipolar affective disorder, current episode mixed	0	0	1	0

F31.7 Bipolar affective disorder, currently in remission	0	0	1	2

F39 Unspecified mood [affective] disorder	0	0	3	0

Notes: No one received a diagnosis of either "F32.3 Severe depressive episode with psychotic symptoms" or "F33.3 Recurrent depressive disorder, current episode severe with psychotic symptoms".

Fourteen patients were diagnosed as psychotic by both CAP and GenP units, providing a total of 76 diagnoses for 62 patients.

Of the one-third (21) of these individuals who received their initial diagnosis of psychosis in connection with CAP care, only two were not considered to have such a condition during later GenP care. The remaining 41 patients were initially diagnosed as psychotic after becoming adults, in connection with GenP care. The overall estimated incidence of first-episode psychosis per 10 000 person-years in our study group was 15.4 (17.1 for females and 13.7 for males).

### Incidence and causes of death by the end of the follow-up period

Three of the 48 patients (6.3%) who were diagnosed with schizophrenia (one with "F21: Schizotypal disorder" and two with "F23: Acute and transient psychotic disorders") had died by the end of the follow-up period, two by suicide and one from a ruptured cerebral aneurysm. This incidence was similar to that among the patients without a diagnosis of schizophrenia.

### Comparison of the patients who were and were not given an ICD-10 diagnosis of schizophrenia and/or psychotic mood [affective] disorder in connection with CAP care

Individuals diagnosed as psychotic in connection with CAP care were older upon initial admission to this care than those without such a diagnosis (p < 0.001 according to the Pearson Chi-Square two-sided test). Furthermore, those with such a diagnosis were more often in need of inpatient CAP care (46.8% versus 19.2%; p < 0.001, Pearson Chi-Square two-sided test); were more often admitted to CAP care primarily for symptoms of anxiety (23.0% versus 12.6%; p = 0.019, Pearson Chi-Square two-sided test); and more often exhibited confusion/disorientation in connection with their initial examination (21.6% versus 0.7%; p < 0.001, Pearson Chi-Square two-sided test). Moreover, all of the patients with a CAP diagnosis of psychosis required continued care in GenP, compared to one-third of those without such a diagnosis.

### Age upon initial diagnosis of psychosis, including a comparison between patients with schizophrenia and psychotic mood disorder

The mean age at the time of initial diagnosis of psychosis among our patients was 21.4 years (range 13–41, SD 6.4) and the corresponding median value was 18.0 years. A majority of these (27 = 44%) were diagnosed between the age of 13 and 17, 17 (27%) between 18 and 25 years of age, 10 (16%) between the ages of 26 and 30 and the remaining 8 (13%) were older at this point in time. A third of those diagnosed as psychotic (21 patients) received this diagnosis in connection with CAP (Table 2) and these patients usually demonstrated more pronounced symptoms. More girls (69.7%) than boys (44.8%) exhibited early onset but this difference was barely statistically significant (p = 0.048, Pearson Chi-Square two-sided). Finally, the 48 individuals diagnosed with schizophrenia were significantly younger (mean age 20.3 years; SD 5.2) at the time of the initial diagnosis of psychosis than were the 12 patients with psychotic mood disorders (mean age 26.8 years; SD 8.3) (p-value: 0.0183, Pearson Chi-Square two-sided test).

**Table 2:** ICD-10 classification of our patients in connection with the initial definitive diagnosis of psychosis

	**Group I**		**Group II**		**Group III**	
***Diagnosis according to ICD-10, Chapter V***	**CAP diagnosis of psychosis**		**GenP diagnosis of psychosis, with certain signs of this condition noted in the CAP record**		**GenP diagnosis of psychosis with no signs of this condition at all noted in the CAP record**	
	**total (n = 21)**		**total (n = 15)**		**total (n = 26)**	
	
***Sub-category***	**N**	**Percentage of total**	**N**	**Percentage of total**	**n**	**Percentage of total**
***Schizophrenia, schizotypal and delusional disorders***	***16***	***76.2***	***13***	***86.7***	***19***	***73.1***

F20 Schizophrenia	1	4.8	7	46.7	5	19.2

F21 Schizotypal disorder	3	14.3	2	13.3	1	3.8

F23 Acute and transient psychotic disorders	12	57.1	2	13.3	7	26.9

F25 Schizoaffective disorders	0	-	2	13.3	4	15.4

F29 Unspecified nonorganic psychosis	0	-	-		2	7.7

***Mood [affective] disorders***	***5***	***23.8***	***2***	***13.3***	***7***	***26.9***

F30 Manic episode	4	19.0	0	-	0	

F31 Bipolar affective disorder	1	4.8	0	-	6	23.1

F 39 Unspecified mood [affective] disorder	0	-	2	13.3	1	3.8

#### The continuity in diagnoses between CAP and GenP care

Of the 531 former CAP patients later admitted to GenP care in adulthood, (38% of the total study population), 20% received a diagnosis within the same ICD-10 category in connection with both types of care, with diagnosis of psychosis at a younger age exhibiting the largest degree of continuity. Thus, of the 27 individuals given such a diagnosis prior to the age of 18, only two were diagnosed differently as adults. One of these received an unspecified diagnosis of anxiety disorder as an adult; while the other, who had been treated for an acute episodic psychosis as an adolescent, was later diagnosed as experiencing some variety of autism.

Of the 21 patients given a diagnosis of psychosis in connection with CAP care, 19 had a psychosis diagnosis in both settings. 12 were placed in the same sub-category of "F20–29: Schizophrenia, schizotypal and delusional disorders" and one in the same sub-category of "F30–39: Psychotic mood disorders" at both time-points. Three patients with a CAP diagnosis in the sub-category of "F20–29: Schizophrenia, schizotypal and delusional disorders" were later categorized as "F30–39: Psychotic mood disorders" in adulthood. In contrast, three individuals treated during adolescence for "F30–39: Psychotic mood disorders" were later categorized in the sub-category of "F20–29: Schizophrenia, schizotypal and delusional disorders".

#### Differences in diagnoses of psychosis between CAP and GenP care

The CAP diagnoses for those 41 patients who were later placed in the categories "F20–29: Schizophrenia, schizotypal and delusional disorders" and/or "F30–39: Psychotic mood disorders" in connection with GenP care are documented in Table 3. Most of these individuals (71%) were treated for problems related to the categories "F90–F98: Behavioural and emotional disorders with onset usually occurring in childhood and adolescence", "F40–F48: Neurotic, stress-related and somatoform disorders" or "Z00–Z99: Factors influencing health status and contact with health services". In addition, three were treated for mental retardation and another three for self-harming behaviour. These patients received their GenP diagnoses at a mean age of 24.0 years (SD 6.35), 19 within 5 years of completion of CAP care, 6 within 6–10 years, 9 within 11–15 years and 7 patients longer than 15 years following discharge from CAP care.

**Table 3:** CAP diagnoses for patients who received a diagnosis of psychosis in connection with GenP care

***Diagnosis according to ICD 10 All***	**Number 41**	**Percentage 100**
F90–F98 Behavioural and emotional disorders with onset usually occurring in childhood and adolescence	15	36.6

F40–F48 Neurotic, stress-related and somatoform disorders	8	19.5

Z00–Z99 Factors influencing health status and contact with health services	6	14.6

F30–39 Mood [affective] disorders (non-psychotic)	3	7.3

F70–F79 Mental retardation	3	7.3

X60–X84 Intentional self-harm	3	7.3

F10–F19 Mental and behavioural disorders due to psychoactive substance use	1	2.4

F50–F59 Behavioural syndromes associated with physiological disturbances and physical factors	1	2.4

F80–F89 Disorders of psychological development	1	2.4

### Information on early signs of psychosis

Of the three different groups of patients that could be discerned, the first and most distinct (Group I) included the 21 (34%) who exhibited signs and symptoms of psychosis in connection with CAP care and, consequently, received their first definitive diagnosis of psychosis as children. Among this group, 14 demonstrated obvious symptoms of a disorder at their initial contact with CAP care-givers, whereas the diagnosis for the 7 others was established only after an observation period of 1–4 years. Accordingly, the mean period of time that elapsed from first admission to CAP care until definitive diagnosis of a psychosis was 2.0 years, (SD 3.6).

A second group (Group II) of 15 patients (24%) also showed possible signs of psychosis during their CAP care, but were first diagnosed with such a disorder in connection with GenP care, mostly at a relatively young age. Their diagnoses were established at a mean of 6.0 years, (SD 5.8) following first admission to CAP care (Table 4, p = 0.0254 compared to Group I; Pearson Chi-Square two-sided test).

**Table 4:** Time period elapsed between completion of CAP care and the first definitive diagnosis of psychosis for patients who received such a diagnosis only in connection with GenP care

**Signs, symptoms, problems, illnesss**	**Time elapsed between completion of CAP care and the initial diagnosis of psychosis**						
	
	2 years or less	3–4 years	5–6 years	7–10 years	11–15 years	16–23 years	Total
	(n = 13)	(n = 6)	(n = 1)	(n = 5)	(n = 9)	(n = 7)	n = 41
***Signs of psychosis noted, all***	***8***	***2***	***0***	***4***	***1***	***0***	***15***

Positive symptoms	7	1	0	3	1	0	12

Cognitive change in attention/concentration	4	0	0	2	0	0	6

Emotional disturbance	2	0	0	0	0	0	2

Negative symptoms	2	0	0	0	0	0	2

Behavioural change	5	2	0	4	1	0	12

Motor/psychical changes	5	0	0	1	0	0	6

***General psychopathology***	***10***	***3***	***0***	***2***	***6***	***5***	***26***

The CAP records for the third group (Group. III) of 26 patients (42%) contained no notation of signs of psychosis and their definitive diagnosis of this disorder was made following completion of the CAP care. For these patients, the period from first admission to CAP to first diagnosed onset of psychosis was even longer, mean 12.4 years, SD 7.9 (Table 4; p < 0.001 compared to Group I and p = 0.0055 compared to Group II, Pearson Chi-square two-sided test). During CAP care, most of this group exhibited unspecific psychopathology, such as behavioural and emotional problems or problems with relationships.

Patients placed in the ICD-10 category "F20–29: Schizophrenia, schizotypal and delusional disorders" demonstrated more symptoms of psychosis at an earlier age than did those classified as "F30–39: Psychotic mood disorders" (p-value: 0.0187 Pearson Chi-square two-sided test).

#### Causes for admission of the patients in Groups I and II to CAP care

The causes for admission of the 36 patients in Groups I and II, who showed signs of psychosis during their CAP care were as follows: confusion or changes in personality (12); free floating anxiety (7); problems with relationships (4); behavioural disorder (3); somatic problems and eating disorders (3); depression (3); suicidal (1); mental retardation and developmental problems (1); pathological reaction to stress (1); and request from a physician for an assessment (1). The symptoms most obviously related to a diagnosis of schizophrenia of some form were confusion or changes in personality (p < 0.001) and free-floating anxiety (p = 0.020).

#### Changes in behavioural

Changes in behaviour e.g., (social isolation, refusal to go to school, loneliness and/or general odd behaviour) were the most frequent first signs/symptoms described in the CAP records of the individuals in Groups I and II who eventually received a F20–29 diagnosis, in connection either with CAP (Group I) or GenP care (Group II). Thirty of these 36 patients (83%) showed such behavioural changes and there was no statistically significant difference between the two groups.

#### Positive symptoms

Signs and symptoms related to schizophrenia were present in 75% of these patients and consisted of: unusual thoughts; bizarre ideas, perceptual abnormalities (such as fear of being poisoned, confusion and suspiciousness) and disorganized speech. Again, there was no significant difference in this respect between Groups I and II.

#### Motor/psychical changes

Both groups contained individuals diagnosed as "F20–29: Schizophrenia, schizotypal and delusional disorders" and "F30–39: Psychotic mood disorders, motor/psychical changes". Signs and symptoms such as motor restlessness, rituals and poor sleep were present in 44% of these patients and equally common among both groups.

#### Cognitive change in attention/concentration

Concentration difficulties and attention deficits, problems with selective attention and forming thoughts, difficulties in comprehension and memory problems were observed in 25% of these cases, somewhat (although not significantly) more often among those classified as schizophrenic. Once again, no difference was found between the groups.

#### Emotional disturbance

31% of the patients in groups I and G II demonstrated impaired emotional functioning or alterations in affect. These features were more frequent among those with psychotic mood disorder and/or belonging to group I, but in neither case were these differences statistically significant.

#### Negative symptoms

Tiredness, listlessness and other negative symptoms were present in 19% of the cases in both Groups I and II, only among those classified as schizophrenic.

#### General psychopathology

The CAP hospital records 67% of those diagnosed with psychotic mood disorders and 60% of those with schizophrenia noted general psychopathology. In 19 of the 36 CAP files for the two groups with early signs (I and II) the symptoms most frequently noted were depression (11 cases), anxiety (8), suicidal intent and self-harm (6) and mania (5). One individual exhibited symptoms of obsessive compulsive disorder and another mood swings.

#### Additional information, signs, symptoms and problems

A variety of additional information concerning the patients in Groups I and II was present in their records, e.g., descriptions of interpersonal difficulties (14 cases), difficulties in relationships with peers (9), stressful life events (12), physical illness (12), a family history of psychosis (7) or of other psychiatric disorder or alcohol abuse in a close relative (9); impaired intelligence (4), low socioeconomic status (3), parents who emigrated to Sweden from another country (3), birth following a complicated pregnancy and/or delivery (3) and a history of neglect/child abuse (2).

### Comparison to an earlier longitudinal study in Sweden

An earlier 20-year prospective follow-up study of 2,164 outpatients (1,417 males and 747 females) discharged from the Stockholm Municipal Child Guidance Clinics in 1953, 1954, and 1955 [4,5] was compared to a sub-group of our own subjects who had been followed-up for a full 20-year period (325 males and 283 females) with respect to the variables described in Table 5. The two groups differed with respect to gender and age distribution, since a larger proportion of pre-school and school boys were included in the Stockholm study. Although the present Jämtland group was older on the averages there were no differences in the frequency of diagnoses in the categories of schizophrenia and psychotic mood disorders. In both groups, the number of patients receiving a diagnosis of psychosis was small. Only 9 individuals in the Stockholm and 6 individuals in the Jämtland groups respectively, were recognized as suffering from a bipolar disorder during a 20 year period of CAP and/or GenP care. Most of the subjects with a diagnosis of psychosis were inpatients.

**Table 5:** Comparison of a subgroup of our outpatients who were followed-up for a full 20-year period with an earlier longitudinal study in Stockholm [4,5]

	**The earlier Stockholm study** **(n = 2.164)**		**The subgroup of our present patients** **(n = 608)**		
	
	**Number**	**Percentage**	**Number**	**Percentage**	p-value*
Males	1,417	65.5	325	53.5	p < 0.001

Females	747	34.5	283	46.5	p < 0.001

Age at the end of the follow up period					

20–31.5 years	1415	65.4	236	38.8	p < 0.001

Older than 31.5 years	749	34.6	372	61.2	p < 0.001

Schizophrenia and or bipolar disorder	30	1.39	17	2.80	n.s.

Schizophrenia	21	0.97	11	1.81	n.s.

Bipolar disorder	9	0.42	6	0.99	n.s.

Note: * Fisher Exact Analysis: Two-sided p-values for p(O> = E|O< = E) (the sum of small p's), n.s. = not significant

## Discussion

As described in the Introduction the present longitudinal prospective study of CAP patients given a diagnosis of schizophrenia and/or of psychotic mood (affective) disorder either in connection with CAP or later GenP care and followed-up for 12 – 28 years after completion of CAP care, was designed to answer a number of specific questions.

Our findings can be summarized as follows: 62 of our 1,400 CAP patients (36 females and 26 males) had received an ICD-10 diagnosis of schizophrenia (48 patients = 3.4% of the total population) and/or psychotic mood disorders (14 = 1%) by the end of the follow-up period. The overall estimated incidence of first-episode psychosis per 10,000 person-years was 17.1. For patients 15–29 years of age, this incidence was lower for males (11.6 versus 16.7) but higher for females (14.2 versus 8.1) than in a study conducted in Australia by Amminger and colleagues [24].

No such gender difference was observed among the 1,338 patients who had not received a diagnosis of psychosis. Three of the 62 patients (4.8%) diagnosed with schizophrenia or mood disorders had died by the end of the follow-up period, two by suicide and one from somatic illness. The corresponding death rate among those without such a diagnosis was similar (2.6%).

The answers to the questions we posed were as follows:

### At what age was the diagnosis of psychosis made?

The mean age of these patients was 21.4 years (range 13–41 years), with 27 (44%) between 13 and 17, 17 (27%) 18–25 and 18 (29%) older than 25 years of age. No other clinical services for psychotic patients in this age range are provided in the geographical area of our study. Females demonstrated an early onset more often than the males.

It is known that a different approach may be required for the early detection and treatment of patients with early-onset psychosis who are more likely to present clinical characteristics associated with a poorer outcome [25,26].

### Was this diagnosis later changed and, if so, in what manner

Only two of the individuals diagnosed as psychotic before the age of 18 years in connection with CAP care did not receive a diagnosis in the category of schizophrenia or psychotic mood disorders as adults. One of them was later diagnosed with an unspecified of anxiety disorder and the other, who was treated for an acute episodic psychosis during adolescence, received a diagnosis in the area of autism. In 13 cases o the CAP diagnoses were later altered in connection with GenP to other diagnoses within the same categories: 12 were placed in the same sub-category of "F20–29: Schizophrenia, schizotypal and delusional disorders" and one in the same sub-category of "F30–39: Psychotic mood disorders" at both time-points. Three patients with a CAP diagnosis in the sub-category of "F20–29: Schizophrenia, schizotypal and delusional disorders" were later categorized as "F30–39: Psychotic mood disorders" in adulthood. In contrast, three individuals treated during adolescence for "F30–39: Psychotic mood disorders" were later categorized in the sub-category of "F20–29: Schizophrenia, schizotypal and delusional disorders".

In their 42-year follow-up of 38 patients with childhood-onset schizophrenia and 38 patients with other diagnoses Remschmidt and co/workers [27] also describe re-diagnosing of former CAP patients as adults. Although their findings do indicate diagnostic stability over time in the case of 91% of their patients, 4 of the individuals (11%) with CAP diagnosis of childhood-onset schizophrenia were given another diagnosis as adults.

Schwartz and colleagues [28] propose that such changes in diagnosis, particularly to schizophrenia, rested primarily on evolution of the illness [28]. Both these investigators and Schimmelmann and co/workers [29] have established the need for a longitudinally based diagnostic process for determining incidences, especially with respect to schizophreniform and bipolar disorders.

### Which early signs of disorder were noted prior or upon admission to CAP care?

Changes in behaviour, including social isolation, refusal to go to school, loneliness and odd behaviour in general were the initial signs/symptoms most frequently observed prior or upon admission to CAP-care. However, this was only the case with regards to the category of schizophrenia. Among the individuals diagnosed with schizophrenia or psychotic mood disorders, symptoms such as motor restlessness, obsessive rituals and poor sleep were equally common, being observed in 44% of the cases. Patients in both of these groups frequently demonstrated anxiety and depression at the time of admission.

### Which patients received their diagnosis later in connection with GenP care and how did this group differ from those diagnosed earlier during CAP care?

The patients given diagnoses of psychoses at an age of 25 years or older exhibited unspecific psychopathological symptoms, but no signs of a possible psychotic disorder during their CAP care. However, the shorter the period that elapsed from the completion of CAP care until admission to GenP care, the more frequently symptoms of a possible psychotic disorder were observed at the CAP unit, although these were not specific enough for a diagnosis to be established.

None of these patients, was diagnosed with childhood-onset schizophrenia, which by definition, debuts before the age 13 [30]. As described by Rapoport and Remschmidt and their colleagues [31-35], this rare disorders is most probably due to progressive brain degeneration and, it therefore is not surprising that none of our 1,400 CAP patients was afflicted.

The scientific literature contains few reports of investigations outside of Scandinavia similar to the present one. In the Nordic countries, findings similar to our own have been reported by Dahl [36] who conducted a 20-year follow-up study of "a child psychiatric clientele with special regard to the diagnosis of psychosis"; by Pedersen and Aarkrog [37,38] who performed a 10- and 20-year follow-up study of child psychiatric patients, and by Strömgren [39] in 1940, when he discussed "Episodic Psychosis in Adolescence". Furthermore, Tyano and co-workers [40] made similar observations concerning "Transient adolescent psychosis" upon monitoring the stability of diagnosis in a cohort of Israeli CAP patients. As discussed above, our current results can be compared to those from a similar 20-year follow-up of child and adolescent psychiatric patients from the 1950's to the 1970's.

#### Limitations of the present investigation

One disadvantage of our present study is that the population of Jämtland County cannot be considered to be representative of the entire Swedish population in all respects. Although comparison with an earlier longitudinal study of outpatients in Stockholm (see above) as well as an unpublished comparison with CAP inpatients in the Stockholm metropolitan area reveals few significant differences, it should be kept in mind that our study group here came from a sparsely populated region. Furthermore, our primary information was obtained from psychiatric hospital records, which are in many respects not scientifically rigorous instruments of examination. Although the quality of these records was considered to be satisfactory, they were assessed employing a protocol chosen for the present study and, moreover, also contain information provided by parents, school personnel and other authorities.

No concurrent validation of the CAARMS extracted from these files employing personal interviews was carried out. The CAARMS instrument, which is basically a manual for personal interview is not intended for interpretation of hospital records. This lack of validation limits our ability to draw conclusions from the signs noted.

In addition, the patients studied here are still relatively young. At the end of our follow-up period, the youngest was 27 years old and had been observed for 12 years, while the oldest was 45 and had been under observation for 28 years. It thus appears likely that additional information of value will emerge from future GenP care concerning the categories "F20–29: Schizophrenia, schizotypal and delusional disorders" and/or "F30–39: Psychotic mood disorders".

## Summary and clinical implications

In summary, it appears that based on empirical findings during the past 70 years, psychotic disorders have been and continue to be relatively uncommon among patients admitted to CAP care in Sweden. The typical male Swedish CAP patient is "a 10-year-old troublesome boy", while the typical female patient is "a 14-year-old depressed girl". Both come from families with psychosocial difficulties; have problems at school and risk later delinquency and/or alcohol and/or drug abuse. However, the rate of schizophrenia observed in our CAP patients (3,4%) is threefold higher than in the general population, indicating that these individuals run an increased risk for developing severe chronic psychosis and that use of a specific treatment model for early psychosis among children and adolescents might be valuable [24].

Our present empirical findings indicate that psychotic disorders debut during the teen-age years and, moreover, that disorders in the ICD category "F20–F29: Schizophrenia, schizotypal and delusional disorders" are more common than those classified as "F30–39: Psychotic mood disorders". Clearly psychotic mood disorders are rare among children and adolescents.

Individuals experiencing early onset of disorders categorized as "F20–F29: Schizophrenia, schizotypal and delusional disorders" may already show typical symptoms upon admission to CAP care at an age of 13–17. In contrast late-onset disorders appear to be very difficult to anticipate on the basis of information gathered in connection with CAP care. In certain of these latter cases, the circumstances necessitating CAP care differed from those leading to later admission to GenP care, when symptoms of a psychotic disorder were apparent. Finally, a handful of our cases appear to have experienced an episodic psychotic disorder during adolescence, as also described previously by Strömgren [39] and more recently by Tyano [40]. For certain of the subjects, in these other two studies, the symptoms described may reflect the first episode of a psychiatric disorder, usually in the category of F20–29, which returns with full force later in life, whereas in other cases the psychosis appears to be episodic.

## Competing interests

The authors declare that they have no competing interests.

## Authors' contributions

UE participated in designing the study, collected the data, performed the statistical analysis and drafted the manuscript. P-AR also participated in the designing study and drafting the manuscript. Both authors have read and approved the final version of this manuscript.
